# The GPR68-NINJ1 axis: an emerging mechano-chemical checkpoint in blood–brain barrier disruption—a hypothetical framework and therapeutic promise

**DOI:** 10.3389/fncel.2026.1757822

**Published:** 2026-03-27

**Authors:** Boren Bai, Haixiao Feng, Huimin Yang, Maokui Huang, Yuechun Wang

**Affiliations:** 1 International School, Jinan University, Guangzhou, Guangdong, China; 2 Gies College of Business, The University of Illinois Urbana-Champaign, Urbana-Champaign, IL, United States; 3 The First Affiliated Hospital of Jinan University, Jinan University, Guangzhou, China; 4 School of Basic Medicine and Public Health, Jinan University, Guangzhou, Guangdong, China

**Keywords:** acidosis, blood–brain barrier, GPR68, mechanobiology, neuroinflammation, NINJ1, therapeutic targeting

## Abstract

The blood–brain barrier (BBB) is a critical interface whose failure is a convergent pathological feature of traumatic, ischemic, and neurodegenerative neurological diseases. Current paradigms often overlook the synergistic interplay between mechanical forces and biochemical cues, such as acidosis, that drive BBB disruption. This perspective synthesizes groundbreaking, yet largely independent, discoveries on two key molecules: GPR68 (OGR1), a proton-sensing GPCR with unique millisecond-level mechanosensitivity to shear stress, and NINJ1, a recently defined executor of plasma membrane rupture during lytic cell death. We propose a testable novel hypothesis: that these proteins form a functional “GPR68-NINJ1 axis,” creating a self-amplifying mechano-chemical circuit that initiates and exacerbates BBB breakdown. We detail the molecular logic of this axis—from GPR68’s sensing of pathological acidosis (pH ≤ 6.4) and shear stress to NINJ1’s oligomerization and DAMP release—and explore its potential role in unifying the pathophysiology of diverse disorders like TBI, stroke, MS, and AD. Finally, we translate this framework into a roadmap for future research and therapeutic intervention, discussing targeted inhibitors, precision chronotherapy, and the critical experiments needed to validate this emerging paradigm.

## 1 Introduction: bridging the mechano-chemical divide in BBB pathology

The blood–brain barrier (BBB) serves as a highly selective physical and biochemical barrier that protects the central nervous system (CNS) by strictly regulating the passage of molecules, ions, and cells between the bloodstream and brain parenchyma, while facilitating essential nutrient transport and waste removal. Paracellularly, tight junctions between brain microvascular endothelial cells (bMECs)—comprising claudin-5, occludin, and ZO-1 scaffolding—seal intercellular spaces, while VE-cadherin-mediated adhesion junctions provide additional mechanical stability. Transcellularly, BBB integrity is preserved by minimal pinocytosis in bMECs, limiting nonspecific transport. Pericyte coverage and astrocyte end-foot ensheathment of the neurovascular unit further regulate permeability and induce barrier properties, collectively ensuring CNS homeostasis (Fong et al., 2024). The dysfunction of BBB is a common pathological endpoint in a spectrum of neurological disorders, including traumatic brain injury (TBI), ischemic stroke (IS), Alzheimer’s disease (AD), and multiple sclerosis (MS) (Archie et al., 2021; Yang et al., 2022; Castillo-González and González-Rey, 2025). While these conditions arise from distinct etiologies—direct mechanical force in TBI, ischemia–reperfusion (IR) in stroke, chronic inflammation in AD, and autoimmune activation in MS—they converge on shared mechanisms of BBB breakdown. A critical, yet underappreciated, driver of this convergence is the synergistic interplay between mechanical forces (e.g., shear stress, physical trauma) and biochemical cues (e.g., tissue acidosis, inflammatory mediators) (Hoffman et al., 2017; Lee et al., 2017; Bonney et al., 2019; Choi et al., 2019; Kuriakose et al., 2019; Nian et al., 2020; Zhao et al., 2023; Konig et al., 2025).

Traditional therapeutic and research paradigms, often siloed by disease-specific approaches, have struggled to address this mechano-chemical synergy (Archie et al., 2021; Sun et al., 2025). While individual pathways are well-studied, their integration into a unified pathological framework remains poorly defined. We posit that bridging this gap requires identifying molecular hubs capable of sensing both physical and chemical insults and translating them into a coherent injury response.

Here, we focus on two such molecules: GPR68 and NINJ1. GPR68 (Ovarian cancer G-protein-coupled Receptor 1, OGR1) is a proton-sensing G protein-coupled receptor (GPCR) that also functions as a rapid mechanosensor for shear stress (Xu et al., 2018). NINJ1 (Nerve Injury-Induced Protein 1), long associated with adhesion and injury, has recently been redefined as the key executor of plasma membrane rupture during lytic cell death, an active process elucidated by cryo-electron microscopy (Kayagaki et al., 2021).

This perspective aims to synthesize fragmented evidence into a testable and novel hypothesis: the existence of a “GPR68-NINJ1 axis” that serves as a central, self-amplifying mechano-chemical checkpoint driving BBB failure. The hypothesis challenges two specific blind spots of this conventional view: (1) the neglect of biomechanical forces (like shear stress) as active, synergistic drivers of injury, and (2) the absence of a unifying framework that integrates diverse physicochemical insults into a common, self-amplifying pathway of terminal cell breakdown. We will outline the molecular basis of this proposed axis, its potential role in unifying neurovascular pathophysiology, and the therapeutic strategies it inspires, while candidly acknowledging the current lack of direct evidence and charting a course for its essential validation.

## 2 GPR68: the putative mechano-chemical sentinel

GPR68 (OGR1) functions as a critical environmental sensor at the neurovascular interface, uniquely capable of detecting and integrating both biochemical and physical danger signals.

### 2.1 Molecular architecture of a dual-sensor

GPR68 is a proton-sensing GPCR activated under pathological acidosis (pH ≤ 6.4), mediated by protonation of key histidine residues (H165/H269) that induces conformational changes and initiates downstream signaling (Ludwig et al., 2003; Matsingos et al., 2024). Its pH-dependent signaling is bidirectional: at pH ≤ 6.4, Gαs-cAMP-EPAC1 activation leads to claudin-5 internalization, while a more severe drop to pH ≤ 6.0 engages the Gαq-PKC pathway, resulting in ZO-1 phosphorylation and tight junction disassembly (Takata et al., 2021; Guo L. et al., 2025).

The functional consequences of this pH-dependent signaling are cell and context-specific, ranging from vasodilation and cytoprotection under mild stress to Ca^2+^ overload and barrier disruption under severe pathological conditions, as summarized in Table 1. This table synthesizes findings from independent studies to propose an integrative model of GPR68’s context-dependent function. The precise thresholds and pathway-outcome pairings represent a theoretical integration based on the available literature and the hypothetical framework of this article. Direct experimental validation of these specific, cell-type-dependent thresholds and their precise mechanistic link to each outcome remains an important area for future investigation.

**Table 1:** Proposed cell and context-dependent signaling of GPR68 and predicted outcomes.

Cellular context and stimulus	Proposed downstream signaling	Predicted functional outcome
Neurons (mild acidosis pH 6.8–7.1/low shear 0.1–2 Pa)	Gq/11-PLC-IP₃/DAG-Ca^2+^-eNOS-NO (Chachisvilis et al., 2006; Zhou and Zha, 2021; Wang et al., 2020; Lim and Harraz, 2024) NF-κB/Hif-1α pathway (Li et al., 2024)	Vasodilation, neuroprotection
Endothelial/microglia (pathological acidosis pH ≤ 6.4/high shear >5 Pa)	Gs-cAMP-EPAC1 signaling Ca^2+^-PKCα-Rap1A (Taylor et al., 2024; Karki et al., 2024)	Endothelial dysfunction, early BBB leakage
Endothelial/immune cells (severe acidosis pH ≤ 6.0/very high shear >10 Pa)	NF-κB/p38 → ICAM-1/VCAM-1↑ → inflammation; VE-cadherin loss → endothelial death (Karki et al., 2024; Karki et al., 2025)	Endothelial death, barrier breakdown

As shown in Table 1, GPR68 also functions as an ingenious mechanosensor (Chachisvilis et al., 2006). Detailedly, GPR68 possesses the ability to sense mechanical stimuli, particularly being sensitive to blood flow shear forces (Xu et al., 2018). Its response to laminar or disturbed flow (0.1–16.7 Pa) is mediated by conformational changes dependent on a cluster of key histidine residues (H17, H20, H84, H169, H269), as evidenced by the significantly impaired shear stress response upon mutation at these sites (Howard et al., 2024). Endothelial GPR68 can be activated by high wall shear stress (WSS) in small-diameter arteries, where it mediates flow-mediated dilation (Xu et al., 2018; Iliff and Xu, 2018). When cells are subjected to shear forces, this mechanical signal is allosterically transmitted through the transmembrane helices to the intracellular G protein-binding domain, leading to the activation of the Gαq/phospholipase C beta (PLCβ) pathway, which breaks down phosphatidylinositol-4,5-diphosphate(PIP_2_) into inositol-3,5-diphosphate (IP_3_) and diacylglycerol (DAG). IP_3_ further induces intracellular calcium reservoirs to release Ca^2+^, thereby increasing intracellular Ca^2+^concentration and leading to downstream signaling events (Ludwig et al., 2003; Lim and Harraz, 2024). In addition, GPR68 responds at millisecond-level (<1 s) sensitivity comparable to ion channels like PIEZO1 (Xiao et al., 2023). This discovery establishes GPR68 as “the only rapid mechanical sensor in the GPCR superfamily”, providing a molecular basis for understanding vascular mechanosensing.

Critically, these sensory modalities are not merely additive but synergistic (Xu et al., 2018; Wei et al., 2018). Acidosis dramatically lowers the receptor’s activation threshold for shear stress. This mechano-chemical integration provides a reasonable molecular explanation for the catastrophic BBB failure observed in IR injury and TBI, where the restoration of blood flow (shear stress) within an acidic environment triggers a disproportionate and devastating injury response that would not occur with either insult alone.

### 2.2 Context-dependent pathological switch

The role of GPR68 is profoundly context-dependent, acting as either a physiological “alarm” or a pathological “amplifier.” Under mild stress or in the ischemic penumbra, GPR68 activation promotes flow-mediated vasodilation and engages cytoprotective pathways, suggesting a neuroprotective role aimed at salvaging compromised tissue (Zhou and Zha, 2021; Wang et al., 2020). However, under the severe acidosis (pH ≤ 6.4) and high shear stress that characterize the ischemic core or the primary impact site in severe TBI, GPR68 signaling shifts decisively to a maladaptive state. Here, it drives sustained Ca^2+^ overload, robust tight junction disassembly, potent neutrophil recruitment, and the initiation of NF-κB-driven inflammatory cascades, ultimately amplifying the initial injury (Long et al., 2023).

## 3 NINJ1: the final executor of membrane integrity

The fundamental understanding of plasma membrane rupture, a terminal event in lytic cell death, has been revolutionized by the recent re-discovery of NINJ1’s role. Once considered a passive, physical consequence of osmotic imbalance, it is now recognized as an active, protein-mediated process executed by NINJ1 oligomers.

### 3.1 A paradigm shift from passive lysis to active rupture

High-resolution cryo-EM structures have provided an atomic-level blueprint of NINJ1’s action, revealing that it assembles into a large, ring-like pore comprising 11–16 monomers. This structural elucidation was a pivotal breakthrough, identifying the extended extracellular loop 2 (ECL2) as the critical oligomerization interface (Degen et al., 2023; Zhu et al., 2025). Furthermore, it pinpointed specific post-translational modifications, such as Lys-69 acetylation, which acts as a regulatory switch by stabilizing the pore (Degen et al., 2023; Yin et al., 2009). This knowledge directly and powerfully enabled the development of targeted therapeutic tools, such as the highly specific monoclonal antibody Clone D1, which was designed to bind the ECL2 interface and sterically hinder pore assembly (Kayagaki et al., 2023).

### 3.2 Cell-type-specific actions in the neurovascular unit

NINJ1’s impact within the neurovascular unit is highly cell-type-specific, reflecting the diverse contributions of different cells to BBB integrity and inflammatory signaling:

*Endothelial cells*: Under resting conditions, NINJ1 expression is minimal. However, inflammatory stimuli (e.g., TNF-α via the JNK/c-Jun pathway) can trigger a dramatic, up to 15-fold induction (Yin et al., 2009; Zheng et al., 2025). Upon activation, NINJ1 oligomerizes into plasma membrane pores, mediating the controlled release of intracellular Damage-Associated Molecular Patterns (DAMPs) like HMGB1 and ATP. These act as potent “danger signals,” fueling a feed-forward inflammatory cycle by recruiting innate immune cells.

*Pericytes*: In mouse models of hypoxia/ischemia, NINJ1 is induced in brain pericytes, where it acts as a negative regulator of angiogenesis by suppressing pro-angiogenic factors such as VEGF and Angiopoietin-1, thereby inhibiting vascular maturation and blood flow recovery (Matsuki et al., 2015; Minoshima et al., 2018). However, direct evidence for NINJ1 expression in human brain pericytes under pathological conditions remains absent. This lack of data represents a critical translational gap, particularly given that transcriptomic analyses reveal divergent gene expression patterns between human and mouse pericytes (Miao et al., 2024), underscoring the limited predictive value of murine models for human cerebrovascular diseases.

*Immune cells*: In neutrophils, NINJ1 is constitutively expressed and mediates the final step of NETosis, the explosive release of chromatin and proteolytic enzymes. The proteases embedded in these Neutrophil Extracellular Traps (NETs), such as elastase and MMP-9, directly degrade tight junctions and the basement membrane (Ahn et al., 2009). In macrophages, NINJ1 operates downstream of the NLRP3 inflammasome and Gasdermin D (GSDMD) activation, executing the final, wholesale mechanical rupture that allows the massive release of inflammatory contents (Kayagaki et al., 2021; Degen et al., 2023; Broz et al., 2020).

NINJ1 thus acts as a convergent pathological executor, the definitive “point of no return” in lytic cell death across multiple CNS cell types.

## 4 A hypothetical framework: the GPR68-NINJ1 axis as a self-amplifying loop

**Figure 1:**
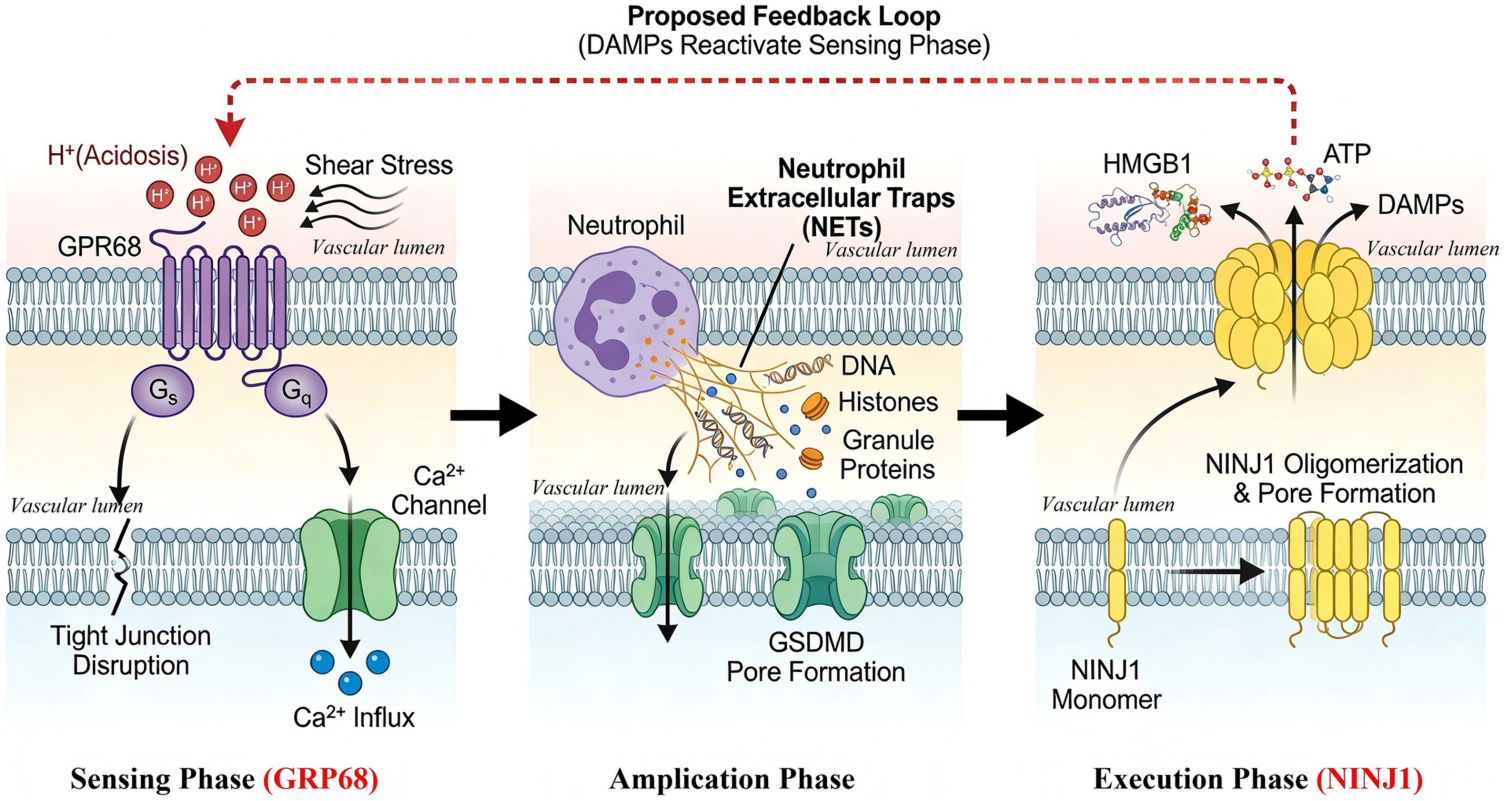
A proposed self-amplifying mechano-chemical loop from GPR68 to NINJ1.

While direct evidence for a physical interaction between GPR68 and NINJ1 is currently lacking, the current established findings from independent studies allow us to propose a functional, self-amplifying module as shown in Figure 1. The Core Molecular Circuit includes:

*Sensing phase*: Pathological acidosis (H^+^) and mechanical shear stress converge to synergistically activate GPR68 on brain endothelial cells.

*Signal transduction & amplification phase*: GPR68 activation triggers rapid Ca^2+^influx, leading to direct disassembly of tight junction proteins (ZO-1, Claudin-5) and the potent transcriptional upregulation of adhesion molecules, facilitating robust neutrophil recruitment.

*The amplification hub*: The recruited neutrophils become a critical nexus, releasing Neutrophil Extracellular Traps (NETs). These NETs, laden with proteases, not only directly damage the vasculature but also induce endothelial pyroptosis, an inflammatory form of cell death, via mechanisms involving TLR4/NF-κB signaling and GSDMD pore formation.

*Execution phase*: The pyroptotic cascade (e.g., caspase-1 activation) serves as a potent trigger for the terminal step: the oligomerization of NINJ1 into plasma membrane pores. This active process dramatically lowers the energy threshold for membrane rupture.

*Feedback loop*: NINJ1-mediated rupture causes the massive release of DAMPs (e.g., HMGB1, ATP) (Kayagaki et al., 2021). These DAMPs further recruit and activate neutrophils (regenerating NETs), exacerbate local acidosis, and potentially generate aberrant mechanical stresses, thereby re-activating GPR68 and closing the vicious cycle (Tadie et al., 2013).

The schematic depicts the proposed feed-forward circuit within the neurovascular unit. The schematic depicts the proposed feed-forward circuit within the neurovascular unit as a vertically aligned but temporally integrated sequence across three phases—Sensing (GPR68), Amplification (Neutrophil/NETs), and Execution (NINJ1)—which should be read from top to bottom.

(The vasculature lumen is labeled to indicate the apical surface where endothelial cells are exposed to blood flow and acidic pH.)

In the autocrine mode, GPR68 activation and NINJ1 oligomerization occur within the same endothelial cell. In the paracrine mode, GPR68-activated endothelium recruit neutrophils and signals to pericytes, triggering NINJ1-mediated lytic death in these neighboring cells and propagating injury across the vascular wall.

Beyond the canonical luminal shear stress mechanism, there is a possibility of additional feed-forward mechanical signals. While GPR68 is primarily activated by luminal shear stress, NINJ1-mediated cell swelling and subsequent tissue edema may generate extravascular mechanical forces—such as compressive or tensile stress—that could theoretically activate GPR68 on abluminal membranes of endothelial cells or on adjacent cell types (e.g., pericytes, astrocytes). While speculative, this feedback mechanism could significantly amplify injury and warrants direct experimental testing in NVU models.

This feed-forward loop provides a reasonable explanation for the rapid, severe, and persistent barrier failure observed in conditions where mechanical and chemical insults coincide, such as TBI and IR injury. The axis is likely engaged in a disease-specific manner: immediately and directly by physical force in TBI; sequentially during reperfusion following ischemia in stroke; via facilitation of immune cell adhesion and transmigration in MS; and through chronic, low-grade activation perpetuated by amyloid and tau pathology in AD.

**Figure 2:**
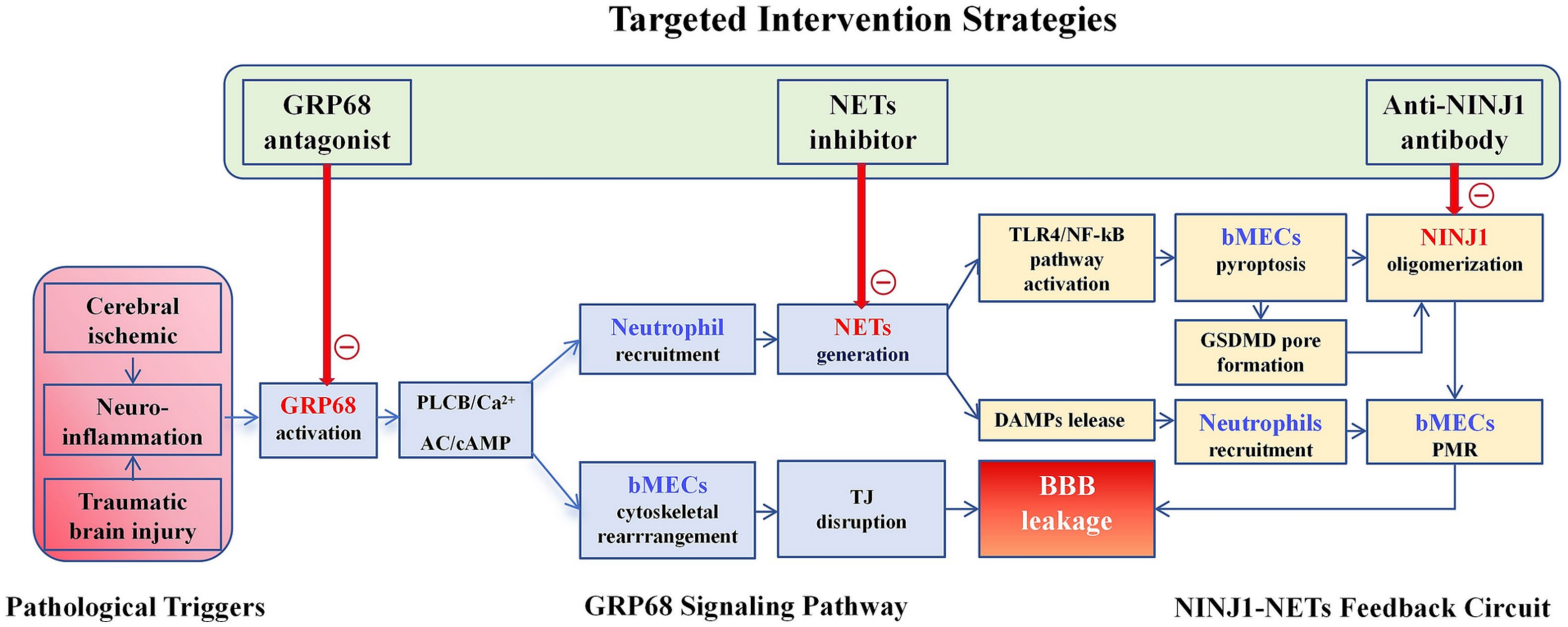
Hypothetical model of the GPR68-NINJ1 axis and intervention strategies in BBB disruption.

As depicted in Figure 2, GPR68 and NINJ1 are not independent actors but form a functionally coordinated “Sensing-Execution” module—the GPR68-NINJ1 axis—that creates a self-amplifying loop driving BBB disruption, which also determines the different intervention strategies as discussed in the coming Section 5.

This schematic integrates the molecular synergy between the sensor (GPR68) and the executor (NINJ1), illustrates the self-amplifying pathogenic loop, and highlights promising therapeutic interventions.

It is important to contextualize this proposed axis within the broader landscape of BBB pathology. NINJ1 is now recognized as a universal executioner of plasma membrane rupture, acting downstream of diverse lethal stimuli, including TNFαand oxidative stress (Xu et al., 2018; Karki et al., 2025). The GPR68-NINJ1 axis is not proposed to be the exclusive pathway to NINJ1 activation. Rather, its unique and critical contribution lies in its role as a primary integrator and amplifier of coincident physicochemical insults. Unlike purely biochemical triggers, GPR68 is uniquely equipped to sense the dual threat of pathological acidosis and shear stress. This positions the axis as a potential initiating trigger in conditions like ischemia–reperfusion injury and TBI, where these forces converge. We hypothesize that GPR68 activation sits at the top of a hierarchical cascade: the initial physicochemical insult activates GPR68, driving endothelial dysfunction and neutrophil recruitment. The subsequent NINJ1-mediated DAMP release can then stimulate paracrine pathways (e.g., TNF/NINJ1) in neighboring cells, which act as downstream effectors to propagate and sustain the injury. Thus, the GPR68-NINJ1 axis may function as the core driver of a self-amplifying loop, while other pathways serve to amplify and perpetuate the initial insult.

## 5 Therapeutic translation: from a conceptual hypothesis to future interventions

The axis framework inspires a new class of therapeutic strategies that target the injury cascade at multiple, complementary points, moving beyond static, single-target approaches.

### 5.1 Targeting the components

*GPR68 modulation*: This requires context-dependent precision. In settings of severe acidosis and high shear stress (e.g., ischemic core, severe TBI contusion), GPR68 antagonists are warranted to block maladaptive signaling, Ca^2+^ overload, and neutrophil recruitment (Karki et al., 2022). Conversely, in milder or chronic settings (e.g., ischemic penumbra), preserving or even enhancing its protective vasodilatory and cytoprotective functions with biased agonists (e.g., Ogerin) could be beneficial (Ludwig et al., 2003; Yu et al., 2019).

*NINJ1 inhibition*: This offers a unique and novel strategy to halt the terminal step of lytic death, effectively “capping” the injury cascade. Monoclonal antibodies like Clone D1 and brain-targeted gene silencing approaches (e.g., using Angiopep-2-modified siRNA nanoparticles) have shown remarkable efficacy in reducing tissue damage and improving functional outcomes in pre-clinical models of stroke and TBI (Habib and Singh, 2022; Guo T. et al., 2025).

### 5.2 Rationale for combinatorial and precision strategies

The feed-forward nature of the GPR68-NINJ1 axis—wherein DAMPs released by NINJ1-mediated rupture reactivate GPR68—provides a mechanistic rationale for why monotherapies may be inadequate: inhibition of the executor alone leaves the sensor active, while blockade of the sensor alone cannot contain the already unleashed DAMP storm. Therefore, disrupting this vicious cycle requires a therapeutic paradigm shift from single-target intervention to strategies that simultaneously intercept both the upstream sensor and the terminal executor.

*Dual-target nanotherapy*: Given the self-amplifying nature of the axis, monotherapies are likely to be insufficient. The co-delivery of GPR68 and NINJ1 inhibitors using advanced nanocarriers (e.g., Angiopep-2-CNPs, iRGD-liposomes) could synergistically disrupt the entire pathogenic loop while simultaneously overcoming the significant challenge of BBB drug delivery (Xia et al., 2024). Endothelial GPR68 is accessible from the luminal side and can be targeted by conventional nanocarriers, while pericyte and neutrophil targets may require actively targeted carriers—such as anti-PECAM-1-functionalized nanoparticles for endothelium or mannose-functionalized liposomes for macrophages—to ensure cell-type-specific engagement and minimize off-target effects.

*Precision chronotherapy*: Early GPR68 agonism is proposed in mildly acidic (pH > 6.4) or normoxic penumbral regions, where protective Gs/NO signaling predominates. Below pH 6.4, GPR68 signaling shifts toward Gq/Ca^2+^-mediated inflammation and barrier disruption. Building on this framework, we now propose a temporally staged intervention strategy that is expected to be refined by injury type, severity, and real-time biomarkers in future translational studies. This model is intended to evolve with emerging biomarker and preclinical data. This involves: early GPR68 agonism (0–4 h) to salvage the penumbra by enhancing perfusion; a switch to GPR68 antagonism (4–24 h) to contain injury once established acidosis sets in; and finally, the introduction of NINJ1 inhibition (>24 h) to resolve the peak wave of membrane rupture and DAMP storm. These windows represent a working model based on preclinical evidence and are expected to be refined by injury type, severity, and real-time biomarkers in future translational studies.

*Next-generation modalities*: Looking ahead, bispecific inhibitors capable of concurrently engaging both GPR68 and NINJ1, and AI-designed, pH-responsive “smart” therapeutics that release their payload or activate only within the pathological acidic microenvironment, represent the vanguard of targeted, context-sensitive neurotherapeutics (Eddy et al., 2025; Yoshida et al., 2024).

## 6 Challenges, validation, and a roadmap for the future

This hypothesis, while built on robust individual discoveries, remains a framework facing significant challenges and requiring direct and rigorous validation.

### 6.1 Acknowledging the hypothetical nature and translational hurdles

The most significant knowledge gap is the precise molecular link between GPR68 activation and the potentiation of NINJ1 oligomerization. Does a specific kinase, a sustained calcium microdomain, or an as-yet-unknown intermediary protein functionally connect the “sensor” to the “executor”?

As discussed in Section 3.1, Lys-69 acetylation stabilizes NINJ1 oligomerization during plasma membrane rupture. GPR68-activated downstream pathways, including PKC (via Gαq/PLC/DAG) and EPAC (via Gs/cAMP), may modulate NINJ1 post-translational modifications (PTMs) by regulating acetyltransferases (e.g., p300/CBP) or deacetylases (e.g., HDACs). Although direct evidence linking GPR68 signaling to NINJ1 PTMs is currently lacking, identifying these PTM dynamics could reveal novel therapeutic targets to disrupt the terminal step of BBB breakdown. It suggests PTM regulation—particularly Lys-69 acetylation of NINJ1—represents a promising mechanistic candidate.

Furthermore, species-specific limitations, particularly the stark contrast in NINJ1 expression and regulation between human and murine pericytes, severely limit the translational predictive value of standard murine models, potentially leading to an overestimation of therapeutic efficacy (Ahn et al., 2009).

Practical delivery bottlenecks also persist, especially for biologic NINJ1 inhibitors like antibodies, where less than 0.2% of the injected dose typically reaches the brain parenchyma, posing a major hurdle for clinical translation (Bajracharya et al., 2021; Kouhi et al., 2021).

### 6.2 A definitive roadmap for validation

The following key experiments are urgently needed to transition this mechanism-based hypothesis from a novel idea to a validated mechanistic paradigm:

*Genetic interaction studies*: Generate and characterize cell-specific (endothelial, pericyte) GPR68/NINJ1 double-knockout models in relevant injury contexts (e.g., stroke, TBI) to dissect their functional interaction and interdependence *in vivo*.

*Human tissue corroboration*: Perform spatial transcriptomics and co-localization studies (e.g., proximity ligation assays) on human post-mortem CNS tissue from patients with TBI, stroke, AD, and MS to determine if the axis components are co-expressed in the same pathological niches.

*Mechanistic linkage*: Employ high-throughput phosphoproteomic and biochemical screens in human endothelial cells and pericytes under acidotic and shear stress conditions to identify the precise signaling molecules (kinases, phosphatases) that functionally link GPR68 signaling to NINJ1 expression or activation. We therefore included phosphoproteomic and acetylome profiling under defined GPR68-activating conditions (acidosis + shear stress) as a high-priority validation step to specifically probe the role of NINJ1 PTM—particularly Lys-69 acetylation—in bridging GPR68 activation to NINJ1 oligomerization.

*Human-relevant modeling*: Utilize advanced human iPSC-derived brain organoids and neurovascular-unit-on-a-chip platforms subjected to controlled acidosis and shear stress to study the axis’s activation and therapeutic inhibition in a human genetic and cellular context, bypassing the limitations of rodent models which remain useful for *in vivo* feasibility but are limited by species-specific differences in NINJ1 regulation and pericyte biology.

*Technological convergence*: Leverage spatial multi-omics to create a high-resolution map of the axis across the NVU; employ AI-driven molecular dynamics simulations to accelerate the design of dual-target inhibitors; and utilize in vivo pH sensors and genetically encoded reporters to empirically validate the proposed spatiotemporal model of axis activation (Chai et al., 2025; Bauer-Rowe et al., 2025).

## 7 Conclusion

The GPR68-NINJ1 axis represents a novel and integrative hypothetical framework for understanding the catastrophic failure of the BBB. While presently a hypothesis, it is built upon a solid foundation of recent structural and mechanistic breakthroughs for both proteins. This perspective aims to catalyze a paradigm shift by focusing the field’s attention on the fertile ground between mechanobiology and inflammatory cell death, proposing a unified pathway through which diverse insults are integrated and amplified. Validating this axis and exploiting its therapeutic potential will require a concerted, multidisciplinary effort that embraces human-relevant models and cutting-edge technologies. If successful, it will not only fill a critical gap in our understanding of neurovascular pathology but also open a new frontier for precise, mechanistically informed, and potentially more efficient interventions for millions of patients with neurological disorders. The journey to validate and target the GPR68-NINJ1 axis constitutes the next compelling challenge in translational neuroscience.

## Data Availability

The original contributions presented in the study are included in the article/supplementary material, further inquiries can be directed to the corresponding authors.
